# Evolution of Chronic Kidney Disease in Different Regions of the World

**DOI:** 10.3390/jcm14124144

**Published:** 2025-06-11

**Authors:** Shoaib Junejo, Mengxuan Chen, Muhammad Usman Ali, Shobha Ratnam, Deepak Malhotra, Rujun Gong

**Affiliations:** Division of Nephrology, University of Toledo College of Medicine, Toledo, OH 43614, USA; shoaib.junejo@utoledo.edu (S.J.); mengxuan.chen@utoledo.edu (M.C.); muhammad.ali2@utoledo.edu (M.U.A.); shobha.ratnam@utoledo.edu (S.R.); deepak.malhotra@utoledo.edu (D.M.)

**Keywords:** chronic kidney disease (CKD), trends, patterns, regional variations

## Abstract

**Background/Objectives:** Chronic kidney disease (CKD) is a major global public health issue, affecting over 690 million individuals worldwide. The prevalence, diagnosis, and treatment of kidney disease vary considerably across different geographical regions. However, comprehensive and in-depth research on CKD remains limited due to its diverse etiologies. **Methods:** This study provides a detailed assessment of the disease burden of CKD, considering its etiological basis and utilizing the latest data reflecting changing trends. This research synthesizes findings from previous studies, with the necessary literature sourced from online platforms such as Google Scholar, EMBASE, and PubMed/MEDLINE databases, as well as Global Burden of Disease (GBD), to compare visualizations of the world’s CKD levels and trends from 1990 to 2021. **Results:** The study results demonstrated that eating patterns are affected by historical and industrial factors, which likely contribute to the high prevalence of CKD in Western countries. The data also indicated that the global number of new CKD cases increased from just over 7.8 million in 1990 to nearly 19 million after 30 years. Additionally, the results showed that age and sex together accounted for the observed prevalence and disease-attributable disability-adjusted life year (DALY) rates in CKD, with the highest rates seen in older age groups, except for CKD attributed to type 1 diabetes, where the incidence was higher in children, and the burden was greater in middle-aged adults. **Conclusions:** Overall, these findings are a valuable addition to the existing literature and provide evidence that CKD studies over a similar time frame reveal notable global trends and regional differences in highlighting this increasing burden.

## 1. Introduction

Chronic diseases are the leading cause of global mortality and morbidity. The rising incidence of chronic kidney disease (CKD) globally, including in developing nations, is attributed to the shift in diseases from communicable to chronic because of financial development, the effects of environmental pollution, and the industrial era [1]. Consequently, CKD has been regarded as a major public health issue since 2017, with approximately 697.5 million cases worldwide being attributable to the aging of populations and population growth. Therefore, CKD is becoming one of the leading causes of death all over the world.

CKD cases vary based on region. For example, as Thurlow et al. stated, in many low-income countries and also some developed nations, high blood pressure and diabetes are the leading causes of CKD [2]. The Asian and sub-Saharan African nations have higher rates of glomerulonephritis and unidentified causes [3]. Furthermore, Stanifer et al. reported that CKD has a significant prevalence [4]. Based on an epidemiological study in Accra, Ghana, it is estimated that nearly 500 million people have CKD, with the majority (80%) of those people living in lower- and middle-income countries (LMICs) [5]. In addition, Paidi et al. determined that CKD was more frequent in older adults and less common in men compared to women [6]. According to Rizzello et al., the diet in Western regions is currently largely related to a high intake of packaged red meat and foods and a low intake of fresh vegetables and fruits [7]. Nevertheless, a comprehensive and detailed analysis owing to various etiologies has rarely been conducted. Therefore, through the use of the most recent data, this research offers a thorough analysis of CKD characterized by various etiologies along with the evolving patterns. The objectives of this study were to identify the pattern of CKD in Western and developing countries, to determine the CKD’s geographic distribution, trends, and regional variation, and to assess the causes of CKD in different regions.

## 2. Materials and Methods

### 2.1. Study Design

The current research adopted a literature review design. According to Snyder, literature reviews are used widely in research investigations to help fill gaps in the existing knowledge of the state of the art of a specific issue [8]. The process of planning when conducting a literature review involves defining the purpose of the study, conducting screening and searching in the literature, data extraction, and executing a review of the literature [9]. Thus, the current research design was appropriate for the literature search to produce a comprehensive overview of CKD stemming from various etiologies and emerging trends based on contemporary data.

### 2.2. Data Source

**Figure 1:**
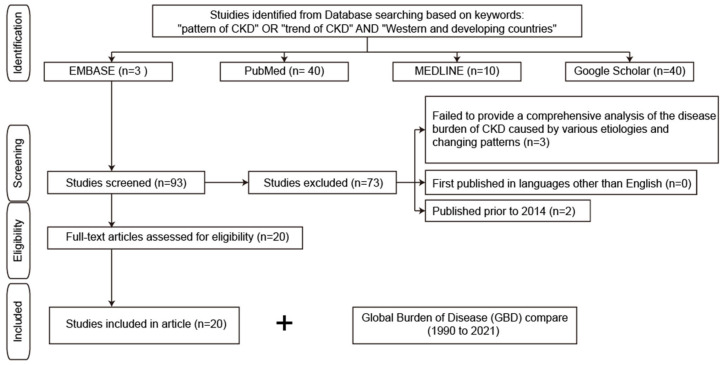
Study search flow diagram. CKD, chronic kidney disease.

The data used in this study were sourced from secondary sources. Secondary data sources are widely used by researchers because they are more easily accessible than primary data sources and are also cheaper and faster to obtain. The published sources were searched from the EMBASE, PubMed, and MEDLINE databases, as well as from the Google Scholar search engine (Figure 1). In addition, Global Burden of Disease (GBD) was used to access visualization of the world’s CKD levels and trends from 1990 to 2021 by accessing the website of the Institute for Health Metrics and Evaluation (IHME) GBD Compare, University of Washington (Seattle, WA, USA), available from http://vizhub.healthdata.org/gbd-compare (accessed on 3 February 2025).

### 2.3. Search Strategy

The study used Boolean operators and search phrases which were designed to align with and support the research objectives. Specifically, the search terms and Boolean operators used to gather data included “pattern of CKD” OR “trend of CKD” AND “Western and developing countries”. Additionally, the investigator ensured that the sources that were included met the subsequent inclusion and exclusion criteria, such as offering a complete analysis of CKD resulting from diverse etiologies and shifting trends utilizing the most recent data to ensure that necessary resources were available.

### 2.4. Inclusion and Exclusion Criteria

The inclusion and exclusion criteria for the literature analyzed in this study are elaborated in Table 1.

### 2.5. Data Analysis

In the current investigation, the themes that were present in the included publications were identified using a thematic analysis approach. For the proposed processes of conducting a thematic analysis on secondary data, Kiger and Varpio highlight key steps starting with familiarization with the data, generating codes based upon the data, forming key themes, analyzing and developing those themes further, and finally reporting on successfully identified themes [10]. Therefore, considering the thematic analysis of the information provided guarantees a thorough examination of whether the employment of the most recent data could help in conducting an analysis of the changing patterns in CKD in different regions.

### 2.6. Ethical Consideration

The main ethical concern in this study was ensuring the responsible use of proprietary information by appropriately citing all referenced works [11]. This was particularly important given that the research relied on the analysis of previously published studies and inquiries. Additionally, the researcher avoided any prejudice by guaranteeing objectivity and impartiality in the sources’ selection and interpretation [11].

## 3. Results

The results, which are based on information from seven publications and GBD comparisons are presented in alignment with the objectives of this study.

### 3.1. Geographic Distribution, Trends, and Regional Variations

#### 3.1.1. Increase in Incidence and Burden

Data from numerous studies have revealed that the prevalence of CKD has increased drastically in the last several decades. Ying et al. found that new CKD cases worldwide had increased from about 7.8 million in 1990 to almost 19 million in 2019 [12].

**Figure 2:**
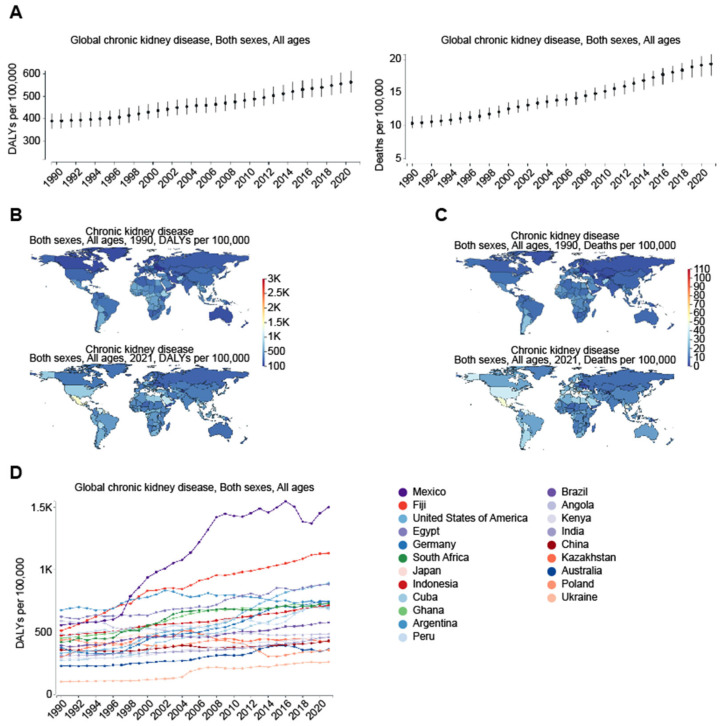
Global increase in chronic kidney disease (CKD) as measures of the global burden of CKD on health comparing the years 1990 and 2021. Figures were generated by Global Burden of Disease (GBD) Compare Data Interactive [https://vizhub.healthdata.org/gbd-compare/ accessed on 1 November 2024], underscoring the increasing prevalence of CKD and its severe impact on health outcomes worldwide. The comparison between 1990 (upper panel) and 2021 (lower panel) further reveals regional variations, illustrating disparities in CKD-related deaths across different regions and income groups. (**A**) Yearly trend in global DALYs and deaths due to CKD from 1990 to 2021. (**B**) Changes in both the DALY rates (per 100,000 population) and their distribution across different regions between the years 1990 and 2021, highlighting trends in mortality and morbidity due to CKD over the three-decade period. DALYs, which combine years of life lost due to premature death (YLLs) and years lived with disability (YLDs), provide a comprehensive measure of the long-term health impact of CKD on populations worldwide. (**C**) Changes in deaths (per 100,000 population) due to CKD between the year 1990 and 2021, emphasizing the growing contribution of CKD to global mortality. (**D**) Yearly trend in DALYs in the indicated countries from 1990 to 2021, with Mexico experiencing the largest increase among all countries over the past three decades.

This estimation was based on the Global Burden of Disease Study 2019 (GBD 2019), including incidence, age-standardized incidence rate (ASIR), disability-adjusted life years (DALYs), and age-standardized DALY rate between 1990 and 2019 by region [13]. On the same issue, Kovesdy (2022) provided supporting views by reporting that more than 800 million people, or over 10% of the world’s population, had CKD, which became worse over time [14]. Consistent with this, the Global Burden of Disease (GBD) comparison (Figure 2A–C) revealed a significant increase in disability-adjusted life-years (DALYs) and deaths due to CKD over the past 30 years globally (Figure 2A). Specifically, DALYs increased from 388.85 per 100,000 population in 1990 to 563.32 per 100,000 population in 2021, while deaths rose from 10.36 per 100,000 population in 1990 to 19.36 per 100,000 population in 2021. Across different regions of the world (Figure 2B), in particular, the DALYs from CKD in the US increased from 330.41 per 100,000 population in 1990 to 892.78 per 100,000 population in 2021. Similarly, this number rose from 555.35 to 1501.4 in Mexico, marking the largest increase among all countries in the past three decades (Figure 2D). In contrast, a more modest increase occurred in China from 356.63 to 430.71, in Taiwan from 458.53 to 867.62, and in Japan from 365.75 to 722.82 per 100,000 population over the same period. Regarding deaths due to CKD (Figure 2C), the US saw an increase from 10.95 per 100,000 population in 1990 to 40.85 per 100,000 population in 2021, while Mexico witnessed an increase from 16.55 to 51.86 during the same period. In contrast, China experienced a slight increase from 8.73 to 14.35, Taiwan from 14.49 to 37.29, and Japan from 14.05 to 41.28 per 100,000 population over the past 30 years. Surprisingly, from 1990 to 2021, some countries, particularly in Africa, saw a decline in CKD. For instance, Ethiopia experienced a reduction in CKD, with the DALYs from CKD decreasing from 922.95 to 481.7 and deaths from CKD dropping from 25.53 to 15.53 per 100,000 population (Figure 2). The underlying causes of this unexpected decline in CKD remain unclear, potentially attributed to the underdiagnosis of CKD, socioeconomic and geographic contexts, less industrialized lifestyles, or unique dietary patterns, which will be analyzed further.

#### 3.1.2. Specific Etiologies

**Figure 3:**
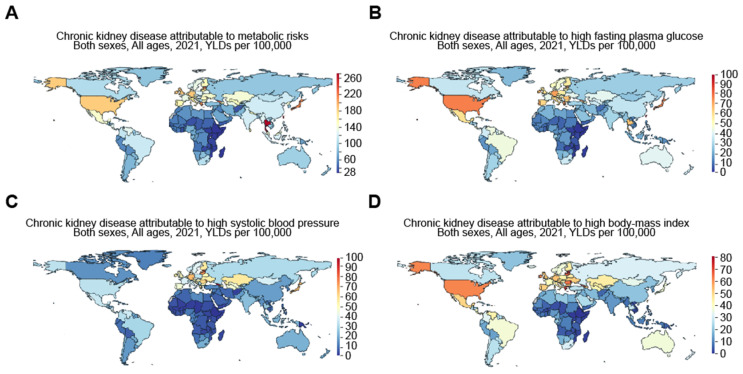
Worldwide increase in CKD attributable to metabolic risks, including high fasting plasma glucose, high systolic blood pressure, and high body mass index. Figures were generated by Global Burden of Disease (GBD) compare Interactive, illustrating years lived with disability (YLDs) in 2021 for CKD attributable to (**A**) metabolic risks, including (**B**) high fasting plasma glucose, (**C**) high systolic blood pressure, and (**D**) high body mass index.

A number of publications have identified certain causes that led to high rates of CKD. The work by Ying et al. [12] backed this up, showing that most of the disease burden was related to CKD due to type 2 diabetes and hypertension. These two causes made up 66% of DALYs and 85% of cases in 2019 for known causes. Additionally, Cockwell and Fisher [15] backed the identified theme, since they concluded that cardiovascular disease or CKD due to poor renal function led to DALYs that differed by more than 15 times between nations, and the highest rates of DALYs were often maintained by nations and regions in the lowest socio-demographic index quintiles. Based on the GBD comparison, the years lived with disability (YLDs) for CKD attributable to metabolic risks in 2021 were 253.68, 118.89, 215.27, 184.47, 144.87, and 28.03 per 100,000 population, respectively, for Taiwan, China, Japan, the US, Mexico, and Ethiopia (Figure 3). More specifically, the YLDs for CKD attributable to high fasting plasma glucose were 98.14, 35.17, 75.27, 76.34, 59.13, and 2.23 per 100,000, respectively, for Taiwan, China, Japan, the US, Mexico, and Ethiopia. Meanwhile, the YLDs for CKD attributable to high systolic blood pressure were 29.41, 18.56, 63.67, 32.84, 34.77, and 3.19 per 100,000, respectively, for Taiwan, China, Japan, the US, Mexico, and Ethiopia, and the YLDs for CKD attributable to a high body mass index were 50.54, 21.43, 39.49, 62.13, 51.06, and 1.48 per 100,000, respectively, for Taiwan, China, Japan, the US, Mexico, and Ethiopia in 2021 (Figure 3).

#### 3.1.3. Notable Changes in ASIR Between the Regions (1990–2019)

Ying et al. [12] backed this theme concept by demonstrating that in North Africa, Central Asia, the Middle East, and Andean Latin America, there were substantial increases in age-standardized incident rate (ASIR). Ying et al. [12] also revealed that there were only slight increases in high-income regions such as Asia Pacific and North America, while other regions showed declining trends, especially for glomerulonephritis-related CKD. To back the above-mentioned theme, an investigation by Kovesdy [14] found significant regional variations by level of income, with the mean age-standardized incidence of CKD in high-income nations being 9.6% and 8.6% for women and men, respectively, and in low- and middle-income countries being 12.5% and 10.6% for women and men, correspondingly.

### 3.2. CKD Causes in Different Regions

#### 3.2.1. Age and Sex Differences

**Figure 4:**
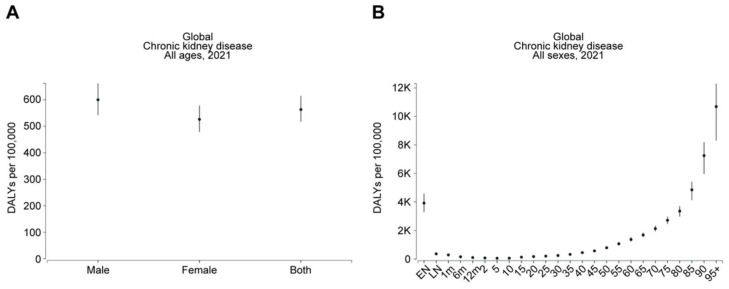
Males and older individuals are at greater risk of CKD. The figures, generated using Global Burden of Disease (GBD) Compare Data Interactive, illustrate the disability-adjusted life-years (DALYs) for CKD, stratified by (**A**) sex and (**B**) age.

An investigation by Ying et al. [12] offered support to this theme by revealing that older people constituted the greatest incidence and DALY rates for CKD, apart from CKD caused by diabetes mellitus type 1, where children showed high incidence rates, while the highest DALY rates were found among those within the middle-age range. Females had a higher ASIR for total CKD, while age-standardized DALY rates for total CKD were highest among males, and specific causes were due to hypertension. Similarly, van Rijn et al. [16] gave supporting views by indicating that the frequency of age-adjusted CKD varied from 7.4% to 13.1%, 7.6% to 13.7%, and 5.5% to 10.4% between nations with high incomes, Eastern and Central European regions, and the remainder of the regions, respectively. Consistent with this, based on the Global Burden of Disease (GBD) comparison, DALYs due to CKD were significantly higher in males (600.09 per 100,000 population) compared to females (526.3 per 100,000 population) in 2021 (Figure 4A). Furthermore, DALYs due to CKD drastically and progressively increased with age. Specifically, DALYs in 2021 were 788.06, 1367.72, 2129.64, 3358.81, and 7252.3 per 100,000 population at ages 50, 60, 70, 80, and 90, respectively (Figure 4B).

#### 3.2.2. Environmental Factors

**Figure 5:**
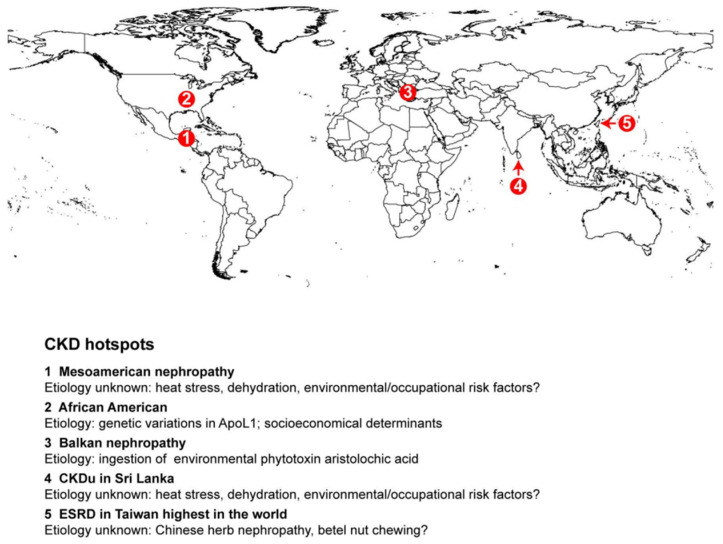
Geographical reginal hotspots for CKD. CKD hotspots are highlighted in specific regions on the global map.

**Figure 6:**
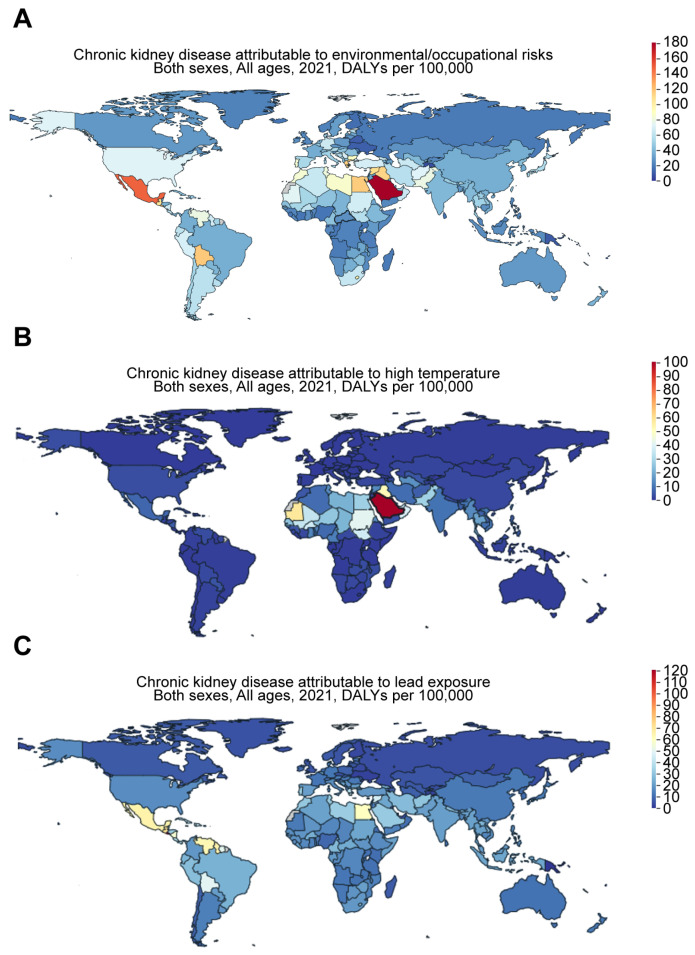
Worldwide increase in CKD attributable to environmental and occupational risks. Figures were generated by Global Burden of Disease (GBD) Compare Data Interactive, illustrating the disability-adjusted life-years (DALYs) for CKD attributable to (**A**) environmental and occupational risks, such as (**B**) high temperatures and (**C**) lead exposure.

Recent evidence suggests that environmental factors contribute to risk of CKD in different regions of the world and may play a role in certain CKD hotspots (Figure 5). For example, CKD of unknown etiology (CKDu) is more likely to occur in tropical areas. Based on the Global Burden of Disease (GBD) comparison, the DALYs for CKD attributable to environmental and occupational risks in 2021 (Figure 6A) were 46.63, 37.06, 56.03, 71.29, 143.94, and 175.69 per 100,000 for Taiwan, China, Japan, the US, Mexico, and Saudi Arabia, respectively. While high temperatures may play a role, their impact appears to be relatively minor. The DALYs for CKD attributable to high temperatures (Figure 6B) were 7.48, 1.89, 0.79, 2.84, 6.49, and 95.42 per 100,000 for Taiwan, China, Japan, the US, Mexico, and Saudi Arabia, respectively. However, exposure to toxic substances, such as heavy metals, may play a more significant role. In support of this, the DALYs for CKD attributable to lead exposure in 2021 (Figure 6C) were 18.48, 13.29, 13.74, 17.33, 63.3, and 31.48 per 100,000 for Taiwan, China, Japan, the US, Mexico, and Saudi Arabia, respectively.

### 3.3. Pattern of CKD in Western Countries

The rising incidence of CKD in Western nations, especially in young, obese females with diabetic kidney disease, is directly related to industrial and historical aspects that have influenced dietary habits, as discussed below.

#### 3.3.1. Historical and Industrial Factors

**Figure 7:**
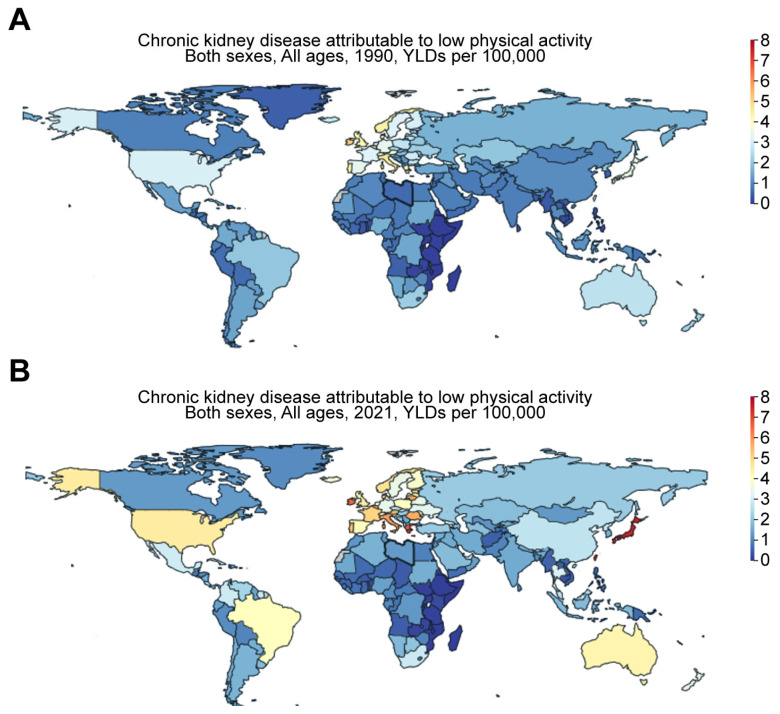
Worldwide increase in CKD attributable to low physical activity. Figures were generated by Global Burden of Disease (GBD) Compare Data Interactive, illustrating years lived with disability (YLDs) in (**A**) 1990 versus (**B**) 2021 for CKD attributable to low physical activity.

Kramer backed up this idea by showing that food habits in Western countries have significantly evolved because of the industrialization of food production and the use of synthetic fertilizer, which boosted farm outputs, leading to higher crop harvests [15]. Crews et al. agreed with this point. They noted that significant disparities arised due to the influence of politics, culture, and economic factors in determining the prevalence of kidney problems and the type of care available across different regions [17]. As a result of industrialization, behavioral changes, such as reduced physical activity, may also contribute to the risk of CKD. The GBD comparison shows that the YLDs for CKD attributable to low physical activity were 7.19, 2.73, 7.31, 4.45, 2.94, and 0.18 per 100,000 population for Taiwan, China, Japan, the US, Mexico, and Ethiopia, respectively, in 2021 (Figure 7). These values were only 3.15, 1.18, 3.53, 3.08, 1.46, and 0.16 per 100,000 population for Taiwan, China, Japan, the US, Mexico, and Ethiopia, respectively, in 1990 (Figure 7). This suggests an aggravating trend in low-physical-activity-associated risk for CKD over the past three decades.

#### 3.3.2. Dietary Patterns and Health Impacts

**Figure 8:**
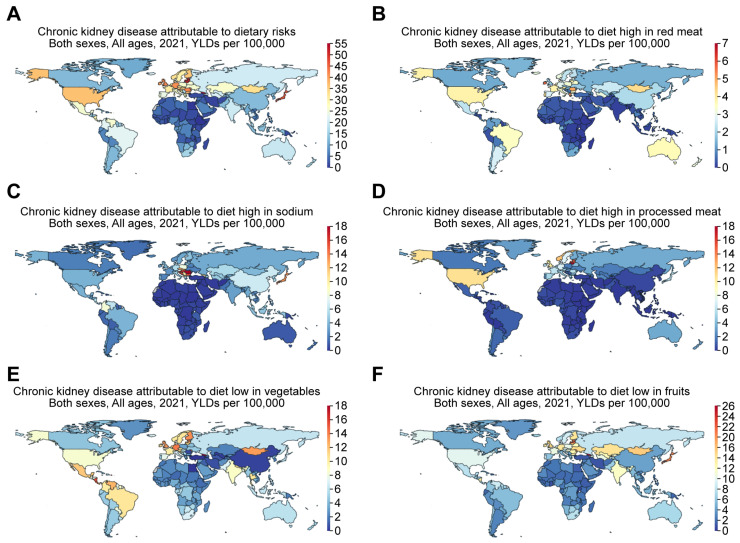
The global increase in CKD is attributed to dietary risks, including diets high in red meat, high in sodium, high in processed meat, and low in vegetables or fruits. Figures were generated by Global Burden of Disease (GBD) Compare Data Interactive, illustrating years lived with disability (YLDs) in 2021 for CKD attributable to (**A**) dietary risks, including (**B**) diet high in red meat, (**C**) diet high in sodium, (**D**) diet high in processed meat, and diet low in (**E**) vegetables or (**F**) fruits.

Kramer [15] established that people in Western countries consume large portions of red meat and processed foods rich in sodium and phosphate, while they consume insufficient amounts of vegetables and fruits. The aforementioned patterns in these diets are associated with increasing obesity trends and related health conditions, including CKD. Choudhury and Kramer also provided more supporting arguments by indicating that in the US, for instance, the mean daily caloric intake increased by around 500 from 2009 to 2019 and could be detrimental to patients with CKD [15,18]. In line with this, the GBD comparison demonstrates that the YLDs for CKD attributable to dietary risks (Figure 8A) were 26.14, 14.75, 44.92, 36.28, 26.57, and 3.16 per 100,000 for Taiwan, China, Japan, the US, Mexico, and Ethiopia, respectively, in 2021. More specifically, the YLDs for CKD attributable to a diet high in red meat were 5.14, 2.17, 1.99, 3.84, 2.39, and 0.046 per 100,000 for Taiwan, China, Japan, the US, Mexico, and Ethiopia (Figure 8B). The YLDs for CKD attributable to a diet high in sodium were 5.64, 6.41, 12.99, 3.76, 3.76, and 0.39 per 100,000 for Taiwan, China, Japan, the US, Mexico, and Ethiopia (Figure 8C). The YLDs for CKD attributable to a diet high in processed meat were 2.31, 0.58, 5.86, 10.75, 1.3, and 0.062 per 100,000 for Taiwan, China, Japan, the US, Mexico, and Ethiopia (Figure 8D). The YLDs for CKD attributable to a diet low in vegetables were 4.34, 0.38, 5.9, 8.02, 10.99, and 1.52 per 100,000 for Taiwan, China, Japan, the US, Mexico, and Ethiopia (Figure 8E). The YLDs for CKD attributable to a diet low in fruits were 6.41, 4.75, 19.89, 10.71, 8.59, and 1.76 per 100,000 for Taiwan, China, Japan, the US, Mexico, and Ethiopia (Figure 8F).

## 4. Discussion

The aim of the current study was to compare the progressive etiology of CKD in Eastern and Western societies and to explore its manifestation in these areas. The following section presents relevant information to the findings outlined above and draws a comparison with conclusions derived from prior research.

### 4.1. Geographic Distribution, Trends, and Regional Variation

This study’s findings revealed that a new CKD incidence of 7.8 million was observed internationally in 1990; it increased drastically to nearly 19 million in 2019. Moreover, other areas, including North Africa, Central Asia, the Middle East, and Andean Latin America, witnessed a rise in the Age-Standardized Incidence Rate (ASIR) within this period. However, the analysis showed further that there was only a marginal rise in high-income countries, including Asia Pacific and North America, whereas other regions depicted actual decline, particularly for CKD with glomerulonephritis etiology. This suggests that since Western areas generally have better access to healthcare and higher categorization in terms of economic status, this would facilitate the early discovery and effective management of CKD because of the availability of facilities. However, access to healthcare services is limited in many developing countries due to financial crises, which suggests that there are differences in CKD trends between Western regions and other emerging countries. In support of the findings of the present investigation, Stanifer et al. identified that most CKD was prevalent in LMICs [4]. This was due to the heightened vulnerability of the population to different types of environmental toxicities, the high prevalence of infectious diseases, and changing epidemiology resulting from rapid urbanization in LMICs. Thurlow et al.supported the findings of this study and demonstrated that the trends in CKD did not change much between 2003 and 2016 in many of the developed countries but increased significantly in the East and Southeast Asia regions [2]. Bello et al. further confirmed these results, indicating global CKD prevalence at a median of 9.5% and a 12.8% increase in incidence rates in Central and Eastern Europe [3]. These results reveal that the trends in CKD usually vary between different geographical distributions and regions.

### 4.2. CKD Causes in Different Regions

The results have shown that CKD is caused by differences in age and sex; older people have the greatest prevalence and DALY rates for CKD with the exception of CKD caused by diabetes mellitus type 1, where incidence rates are higher in children, and DALY rates are higher in middle-aged people. The findings imply that given that the kidneys’ capacity for filtering waste products from the blood declines with age, there is an increase in the risk of developing CKD, and as a result, its prevalence is higher in older people. Additionally, it was determined that males show higher age-standardized DALY rates for both total CKD and individual causes, particularly CKD caused by hypertension, whereas females have a higher ASIR for total CKD. These outcomes suggest that, in general, women are more likely than men to have CKD due to biological variations, like the effects of hormones on renal function. In support of the results above, Thurlow et al. [2] revealed that the US population’s anticipated clinical, lifestyle, and demographic traits could potentially reverse the current declining trend. However, research by Paidi et al. contradicted these assertions by demonstrating that CKDu predominantly affects young and middle-aged individuals, with a slight male predominance, and is not associated with traditional risk factors such as hypertension and diabetes [6]. Nevertheless, with regard to the above discussion, one can infer that the patterns in CKD typically vary across different countries and geographical distributions for a variety of reasons, including changes in sex and age.

A study conducted by Agyei-Mensah and Aikins in 2010 [5] delves into the health transition in Accra, Ghana, underscoring the dual burden of infectious and chronic diseases. Wealthier regions predominantly encounter chronic diseases, while impoverished areas grapple with both infectious and chronic ailments. Urbanization, poverty, and globalization are pivotal factors driving these transformations.

### 4.3. The Pattern of CKD in Western and Developing Countries

The results showed that the historical and industrial aspects that have impacted dietary patterns are directly contributing to the rising incidence of CKD in Western countries, especially among young and morbidly obese women with diabetic kidney disease. The United States’ obesity epidemic reflects larger shifts in food production and consumption, which have important health ramifications. These results imply that provided that uranium, cadmium, and lead are among the heavy metals frequently found in synthetic fertilizers, they have the potential to build up in the environment and make their way into the food chain and interfere with renal function and, as a result, can harm the kidneys when consumed. Nonetheless, findings on the use of antibiotics imply that they have the potential to directly harm kidney cells during filtration or produce crystals that obstruct urine flow, and the resistance brought on by overuse can make infections more difficult to cure and perhaps exacerbate renal problems. An investigation by Rizzello et al. [7] provided supporting insights by noting that the US or Western diet is characterized by a low consumption of fresh vegetables and fruits and a high intake of processed foods and red meat preserved with phosphate and salt. As such, consuming more animal protein, salt, and processed foods preserved with inorganic phosphate salts is typically associated with a greater influence in causing CKD. Furthermore, the incidence of certain CKD has disproportionally increased in the past several decades. For example, in the 1970s and early 1980s, focal segmental glomerulosclerosis (FSGS) accounted for 10% to 15% of cases of idiopathic nephrotic syndrome in adults. However, there was an approximately sevenfold increase in the incidence of FSGS from 1974 to 1993 in an active renal biopsy practice [19]. Therefore, from these discussed findings, it can be concluded that CKD in Western nations has continued to exacerbate due to various factors, including high food intake and dietary patterns.

Chronic kidney disease (CKD) poses a significant health challenge in low- and middle-income countries (LMICs), where it is the 12th leading cause of death worldwide [4], with an 82% increase in mortality since 1990 [20]. These regions experience high CKD prevalence due to rapid population growth and the heavy impact of non-communicable diseases (NCDs) [20]. In Asia, CKD rates are notably high, ranging from 10% to 20% in countries such as China, Thailand, Malaysia, Bangladesh, India, Pakistan, Nepal, and Sri Lanka. Similarly, sub-Saharan Africa reports a CKD prevalence between 5% and 17% [3], hindered by low awareness and limited access to treatment [4]. In Latin America, urban and rural areas face significant CKD burdens, with Mexico and Central American agricultural communities reporting high rates, often linked to diabetes and obesity [4]. The Middle East also faces a significant CKD burden, with diabetes as a major contributor; for instance, Iran reports prevalence rates up to 23%, and Turkey at 16% [20]. The diverse causes of CKD in LMICs reflect rapid urbanization and lifestyle changes, such as increased hypertension and diabetes, compounded by environmental factors like poor infrastructure and pollution. However, limited research and inconsistent methodologies represent obstacles in acquiring a full understanding of CKD epidemiology. The lack of longitudinal studies complicates the establishment of causal relationships, such as the role of hypertension in CKD [4].

Kidney disease in Latin America presents a significant health burden, with both chronic kidney disease (CKD) and acute kidney injury (AKI) being prevalent [21]. In low-income countries, CKD risk factors include low birth weight and infections, whereas middle-income countries share similar causes with developed nations. The prevalence of end-stage renal disease (ESRD) has risen markedly, from 119 patients per million population in 1991 to 669 in 2013, with a mix of patients on hemodialysis, those on peritoneal dialysis, and those with kidney transplants [22]. Countries like Puerto Rico, Argentina, Brazil, Uruguay, Chile, and Mexico report the highest rates of ESRD, highlighting the need for effective healthcare strategies to manage this increasing burden [22].

A unique aspect of chronic kidney disease (CKD) epidemiology in some Latin American countries is the emergence of CKD of unknown origin (CKDu), also known as Mesoamerican nephropathy. This condition is prevalent among young male agricultural workers who perform strenuous labor in extreme heat. Unlike typical CKD, CKDu is not linked to diabetes or hypertension. The primary suspected cause is recurrent dehydration due to heat stress, though other factors such as exposure to agrochemicals, pesticides, nonsteroidal anti-inflammatory drugs, heavy metals, infectious agents like hantavirus or leptospira, and other nephrotoxins may also contribute [23].

In countries like Guatemala, CKDu is a leading cause of premature death among young adult men. Clinically, it resembles tubulointerstitial diseases, with features like normal or mildly elevated blood pressure, hyperuricemia, hypokalemia, and hyponatremia. There is often microalbuminuria but no significant proteinuria. Urine microscopic examination is typically unremarkable, and renal ultrasounds show nonspecific findings such as small kidneys and a loss of normal corticomedullary differentiation. Renal biopsies from CKDu patients frequently reveal tubular atrophy and interstitial fibrosis, with no vascular lesions or mesangial proliferation. Some glomerular changes, the thickening of the Bowman’s capsule, and the shrinking of glomerular capillaries indicate potential renal ischemia [24].

Regarding the prevalence of chronic kidney disease (CKD) in China, a nationally representative cross-sectional study conducted from 2018 to 2019, as part of the Sixth China Chronic Disease and Risk Factor Surveillance, estimated that approximately 8.2% of Chinese adults, or around 82 million people, had CKD. This study noted a 30% decrease in CKD prevalence over the past decade, attributed to better environmental protection, the integration of CKD into national public health surveillance, and the control of common comorbidities such as hypertension and diabetes [25]. In contrast, data from the Global Burden of Disease (GBD) 2019 reported a higher prevalence, with an estimated 150.5 million cases of CKD in China in 2019, accounting for 10.6% of the population. This study highlighted an increasing trend in CKD prevalence and mortality over the past three decades, with significant age, period, and cohort effects [26].

Another study involving 9.5 million adults from the Meinian Onehealth screening survey in 2017 reported a lower prevalence of 1.07% after adjusting for urban and rural areas, suggesting around 14 million Chinese adults with CKD [26]. This study also found a higher CKD prevalence in older adult females and certain geographic regions, with significant associations between CKD and risk factors such as hypertension, cardiovascular disease, diabetes, and metabolic parameters [27]. In addition, it has been reported that the prevalence of glomerular diseases in China has shifted, with the adjusted odds for membranous glomerulopathy increasing by 13% annually from 2004 to 2014. A potential cause of this increased risk is linked to particulate matter 2.5 (PM_2.5_), a type of air pollution consisting of extremely small particles that can be inhaled [28] and subsequently elicits oxidative stress and upregulates PLA2R expression in the lungs, resulting in the pathogenesis of membranous nephropathy through extracellular vesicles [28]. Overall, while the exact prevalence figures vary, all studies agree that CKD is a significant public health concern in China, with a need for continued public health strategies to detect and reduce modifiable risk factors to mitigate the disease burden.

Taiwan faces a significant public health challenge due to its exceptionally high incidence and prevalence rates of end-stage renal disease (ESRD) and chronic kidney disease (CKD). This region has the highest incidence of ESRD globally (Figure 5), with diabetes mellitus (43.2%), chronic glomerulonephritis (25.1%), hypertension (8.3%), and chronic interstitial nephritis (2.8%) being the major underlying causes of ESRD [27,29,30]. The prevalence of CKD in Taiwan is substantial, with estimates indicating 6.9% for CKD stages 3–5, 9.83% for clinically recognized CKD, and 11.9% for CKD stages 1–5. However, the overall awareness of CKD remains low, at 9.7% for stages 1–3 and 3.5% for stages 1–5 [29,30]. Risk factors for CKD include older age, diabetes, hypertension, smoking, obesity, the regular use of herbal medicine, family history, chronic lead exposure, and hepatitis C. Chinese herbal therapy, particularly products containing aristolochic acid, is also a significant risk factor [31]. In addition, betel nut chewing is common in Southern China, including Taiwan, and has been identified as a risk factor for chronic kidney disease (CKD) independent of factors such as age, BMI, smoking, alcohol consumption, hypertension, diabetes, or hyperlipidemia [32].

A family history of ESRD is also a significant risk factor, with individuals having a first-degree relative with ESRD being 2.5 times more likely to develop ESRD. Genetic and environmental factors contribute to the development of ESRD, with heritability estimated at around 31.1% and nonshared environmental factors accounting for 57.5% of the phenotypic variance [27].

The findings of this study provide important information to the stakeholders in charge of monitoring CKD to help clarify information already available on its global trends and variations between developed Western countries and emerging ones such as in Sub-Saharan Africa. Lastly, the research’s findings will be useful for subsequent researchers since they will add to the scholarly discourse on the subject of the trend in CKD across various countries.

This study was limited by the small number of sources that used qualitative, quantitative, or combination approaches that were found as a result of using the literature review approach. Finally, the fact that the data used in the present research were sourced solely from the existing literature presents another significant limitation. This restriction indicates that the perspectives of both the investigator and the participants regarding the issues under investigation were not considered.

### 4.4. The Potential Impact of Migration on Future Projections of CKD Prevalence

Migration and chronic kidney disease (CKD) present unique challenges that necessitate tailored public health strategies to effectively address CKD in migrant populations. Several factors influence the relationship between migration and CKD. Lifestyle changes associated with urbanization and migration to high-income regions can increase exposure to CKD risk factors like obesity and hypertension [33]. Access to healthcare is a significant determinant, as migrants often face barriers in the early detection and management of CKD [33]. The “healthy immigrant effect” suggests that immigrants initially experience better health outcomes, including lower CKD risk, compared to native populations [34]. However, the health of immigrants declines with longer residence in the new country, potentially increasing CKD risk over time [35,36]. Kidney function declines similarly in US-born and foreign-born individuals living in the US for 15+ years.

## 5. Conclusions

The current study sought to conduct a thorough analysis of CKD resulting from various etiologies and evolving trends, utilizing the most recent data. Based on the results that were presented in the previous section, it is appropriate to conclude that the first study’s objective was appropriately addressed, with the inference that the increasing incidence of CKD in Western nations is largely attributable to historical and industrial factors that have affected eating patterns, particularly among young, morbidly obese women with diabetic kidney disease. Nevertheless, the results presented additionally tackled and evaluated the second objective of the current study by showing that around 7.8 million incident instances of CKD occurred globally in 1990, whereas by 2019, that number had risen to nearly 19 million. The findings also show that the third objective was effectively met, which led to the conclusion that age and sex disparities contribute to the prevalence and DALY rates of CKD, with older adults having the highest rates, with the only exception being CKD caused by type 1 diabetes, where the prevalence rates were greater in children, and DALY rates were higher in middle-aged adults. In summary, the findings of this study add to the body of literature and support the claim that through the thorough examination of CKD from 1990 to 2019, we can identify notable worldwide patterns and variations that highlight both the growing burden and regional differences.

## 6. Perspectives

The research design chosen for this study permits the replication of similar studies in the future by increasing the sample size, allowing for more reliable results. This is accomplished by combining information from a greater variety of sources. Additionally, potential researchers could use strategies that allow for the gathering of primary data from participants in relation to the research questions, for instance, patients with CKD and healthcare providers in various nations, to extract their individual viewpoints and opinions with regard to inquiries to evaluate the present study’s goal.

## Figures and Tables

**Table 1:** Inclusion and exclusion criteria for the literature analyzed in this study.

Inclusion Criteria	Exclusion Criteria
Research offering a complete analysis of the disease burden of CKD resulting from diverse etiologies and shifting trends, utilizing the most recent data.	Articles that failed to provide a comprehensive analysis of the disease burden of CKD caused by various etiologies and changing patterns were excluded.
	Articles first published in languages other than English were disregarded.
Articles published in the last ten years.	Studies published prior to 2014 were excluded as they were considered outdated.

## Data Availability

There are no data associated with this study beyond what is published in the main text. There are no template data collection forms associated with this study, as data were extracted directly from http://vizhub.healthdata.org/gbd-compare.
